# Cantharidin represses invasion of pancreatic cancer cells through accelerated degradation of MMP2 mRNA

**DOI:** 10.1038/srep11836

**Published:** 2015-07-02

**Authors:** Meng Shen, Meng-Yao Wu, Long-Pei Chen, Qiaoming Zhi, Fei-Ran Gong, Kai Chen, Dao-Ming Li, Yadi Wu, Min Tao, Wei Li

**Affiliations:** 1 Department of Oncology, the First Affiliated Hospital of Soochow University, Suzhou 215006, China; 2 Department of General Surgery, the First Affiliated Hospital of Soochow University, Suzhou 215006, China; 3 Department of Hematology, the First Affiliated Hospital of Soochow University, Suzhou 215006, China; 4 Jiangsu Institute of Clinical Immunology, Suzhou 215006, China; 5 Institute of Medical Biotechnology, Soochow University, Suzhou 215021, China; 6 PREMED Key Laboratory for Precision Medicine, Soochow University, Suzhou 215021, China

## Abstract

Cantharidin is an active constituent of mylabris, a traditional Chinese medicine, and is a potent and selective inhibitor of protein phosphatase 2A (PP2A) that plays an important role in cell cycle control, apoptosis, and cell-fate determination. In the present study, we found that cantharidin repressed the invasive ability of pancreatic cancer cells and downregulated matrix metalloproteinase 2 (MMP2) expression through multiple pathways, including ERK, JNK, PKC, NF-κB, and β-catenin. Interestingly, transcriptional activity of the MMP2 promoter increased after treatment with PP2A inhibitors, suggesting the involvement of a posttranscriptional mechanism. By using an mRNA stability assay, we found accelerated degradation of MMP2 mRNA after treatment of cantharidin. Microarray analyses revealed that multiple genes involved in the 3'→5' decay pathway were upregulated, especially genes participating in cytoplasmic deadenylation. The elevation of these genes were further demonstrated to be executed through ERK, JNK, PKC, NF-κB, and β-catenin pathways. Knockdown of PARN, RHAU, and CNOT7, three critical members involved in cytoplasmic deadenylation, attenuated the downregulation of MMP2. Hence, we present the mechanism of repressed invasion by cantharidin and other PP2A inhibitors through increased degradation of MMP2 mRNA by elevated cytoplasmic deadenylation.

Pancreatic cancer is one of the deadliest solid malignancies, with a 5-year survival rate of only approximately 5%. There is still no method for early detection of pancreatic cancer, and most patients with localized cancer have no recognizable symptoms. As a result, most patients are not diagnosed until after the cancer has metastasized to other organs^1^. Less than 20% of patients are eligible for curative resection, and of those, most experience recurrence of the cancer. Thus, an effective treatment and therapy are essential^2^.

Mylabris is the dried body of the Chinese blister beetle. The use of mylabris as a traditional Chinese medicine in the treatment of tumours can be traced back to more than 2000 years ago, and it is still being used as a folk medicine today^3^. The active constituent of mylabris is cantharidin^3^. In our previous studies, we found that cantharidin presented cytotoxicity against pancreatic cancer cells through the NF-κB pathway that mediates apoptosis induction^4^, the c-Jun N-terminal kinase (JNK) pathway that inhibits cell growth^3^,^5^, and the Wnt/β-catenin pathway that inhibits cell migration^6^. A recent study reported that cantharidin could also repress the invasion of bladder carcinoma cells through the downregulation of matrix metalloproteinase 2 (MMP2)^7^, the main proteinase that participates in the degradation of cellular matrix. However, the detailed mechanism involved still requires full exploration. In the present study, we investigated whether cantharidin could repress the invasive ability of pancreatic cancer cells through downregulation of MMP2.

Mechanistically, cantharidin is a selective inhibitor of serine/threonine protein phosphatase 2A (PP2A). In our previous studies, we observed that inhibition of PP2A in pancreatic cancer cells resulted in phosphorylation of multiple substrates, including extracellular signal-related kinase (ERK), JNK, IκB kinase (IKK), protein kinase C (PKC), and β-catenin. Therefore, we looked to determine if these pathways were involved in the regulation of invasion and MMP2 expression by cantharidin in pancreatic cancer cells.

## Materials and Methods

### Cells and cultures

The human pancreatic cancer cell lines, PANC-1 and CFPAC-1, were purchased from the American Type Culture Collection (ATCC, Manassas, VA, USA). Cells were maintained in DMEM medium (Gibco, Grand Island, New York, USA). Medium was supplemented with 10% fetal calf serum (Gibco), 100 units/mL penicillin, and 100 mg/mL streptomycin at 37 °C in a 5% CO_2_ incubator with humidified atmosphere. Cells were passaged every 2–3 days for exponential growth.

### Reagents

Cantharidin, Okadaic acid (OA), prostaglandin E2 (PGE2), PD98059, SP600125, RO31-8220, and GF109203X were purchased from Enzo Life Science International (Plymouth Meeting, PA, USA). Norcantharidin (NCTD), Bay11-7082, EF-24, and actinomycin D (ActD) were purchased from Sigma (St. Louis, MO, USA).

### Invasion assays

A total of 100 μl of Matrigel (1:30 dilution in serum-free DMEM medium) was added to each Transwell polycarbonate filter (8-μm pore size; Corning, NY, USA) and incubated with the filters at 37 °C for 6 hours. Cells were trypsinized and washed three times with DMEM medium containing 1% FBS, followed by resuspension in DMEM containing 1% FBS at a density of 2 × 10^6^ cells/ml. The cell suspensions (100 μl) were seeded into the upper chambers and 600 ml of DMEM medium containing 10% FBS was added to the lower chambers. Cells (2 × 10^5^/well) were allowed to invade for 12 hours and membranes were then stained with 1% methylrosanilinium chloride. Cells that had migrated to the underside of the filter were counted using a light microscope in five randomly selected fields.

### Real-time PCR

Total RNA was extracted using Trizol reagent (Invitrogen, CA, USA) according to the manufacturer’s protocol. After spectrophotometric quantification, 1 μg total RNA in a final volume of 20 μl was used for reverse transcription with PrimeScript RT Reagent Kit (TaKaRa, Otsu, Shiga, Japan) according to the manufacturer’s protocol. Aliquots of cDNA corresponding to equal amounts of RNA were used for quantification of mRNA by real-time PCR using the LightCycler 96 Real-time Quantitative PCR Detection System (Roche, Indianapolis, IN, USA). The reaction system (25 μl) contained the corresponding cDNA, forward and reverse primers, and SYBR Green PCR master mix (Roche). All data were analyzed using B2M gene expression as an internal standard. The specific primers are presented in Table 1.

### Western blot

Mouse anti-MMP2, mouse anti-PP2Ac, and mouse anti-B2M antibodies were purchased from Santa Cruz Biotechnologies (Santa Cruz, CA, USA). Total protein was extracted using a lysis buffer containing 50 mM Tris–HCl (pH 7.4), 150 mM NaCl, 1% Triton X-100, 0.1% SDS, 1 mM EDTA, and supplemented with protease inhibitor cocktail kit (Roche). The protein extract was loaded onto an SDS-polyacrylamide gel, size-fractionated by electrophoresis, and then transferred to polyvinylidene fluoride (PVDF) membranes (Bio-Rad Laboratories, CA, USA). After blocking in 5% non-fat milk for 1 hour, the membranes were incubated overnight with primary antibodies at 4 °C. The protein expression was determined using horseradish peroxidase-conjugated antibodies followed by enhanced chemiluminescence (ECL, Millipore, St Charles, MO, USA) detection. The intensity of the bands was captured by JS-1035 image analysis scanning system (Peiqing Science & Technology, Shanghai, China). B2M was used as the internal control.

### Zymography

Cells (1 × 10^5^) were seeded onto wells of 24-well plates and allowed to adhere in the presence of serum. Media was subsequently replaced by 0.5 mL of serum-free medium per well. After 24 hours of incubation, the conditioned media was harvested for zymography. A total of 25 μl of the conditioned medium for each sample was subjected to 10% SDS/PAGE with 1 mg/ml gelatin incorporated into the gel mixture. Following electrophoresis, the gels were soaked in 2.5% Triton X to remove SDS, rinsed with 10 mmol/l Tris (pH 8.0), and transferred to a bath containing 50 mmol/l Tris (pH 8.0), 5 mmol/l CaCl_2_, and 2 μmol/l ZnCl_2_ at 37 °C for 17 hours. The gels then were stained with 0.1% Coomassie blue in 45% methanol and 10% acetic acid.

### Knockdown of target genes

Target specific small interfering RNAs (siRNAs) were designed and synthesized by Genepharma (Shanghai, China). The specific sequences were as follows: (1) Control-siRNA, sense, 5′-UUCUCCGAACGUGUCACGUdTdT-3′, anti-sense,5′-ACGUGACACGUUCGGAGAAdTdT-3′; (2) PP2Acα-siRNA, sense, 5′-CGUGCAAGAGGUUCGAUGUdTdT-3′, anti-sense, 5′-ACAUCGAACCUCUUGCACGdTdT-3′; (3) MMP2-siRNA, sense, 5′-GGAGAGCUGCAACCUGUUUdTdT-3′, anti-sense, 5′-AAACAGGUUGCAGCUCUCCdTdT-3′. The transfections were performed with siRNA-Mate Transfection Reagent (Genepharma) according to the manufacturer’s protocol. Lentivirus gene transfer vectors (LV2-pGLV-u6-puro) containing small hairpin RNA (shRNA) of target genes were constructed by Genepharma and confirmed by sequencing. The specific sequences were as follows: (1) Control, 5′-TTCTCCGAACGTGTCACGTTTC-3′; (2) CNOT7-1, 5′-GCAAGACCCATTGGAGAATTC-3′; (3) CNOT7-2, 5′-GGAGAATACCCTCCAGGAACT-3′; (4) PARN-1, 5′-GGAAGTATCCGAAAGGCATTC-3′; (5) PARN-2, 5′-GCAGCAGAAACATGCCAAAGA-3′; (6) RHAU-1, 5′-GCATTACCATAGATGATGTCG-3′; (7) RHAU-2, 5′-GGAGTCCACTTGGCACGATTA-3′. The recombinant lentivirus was prepared and titered to 3 × 10^8^ TU/ml (transfection unit).

### Luciferase reporter gene assay

The reporter plasmid, pGL2-MMP2, which contains a 1,716-bp DNA fragment (bp -1659 to 157) upstream from the transcription initiation site of the *MMP2* gene^8^ was kindly provided by Yi Sun (Department of Molecular Biology, Parke-Davis Pharmaceutical Research). The internal control plasmid, pRL-SV40, which contains the renilla *luciferase* gene, was obtained from Promega (Madison, WI, USA). Cells were transiently cotransfected with the reporter plasmid (500 ng/well) and the pRL-SV40 plasmid (100 ng/well) for 8 hours using Lipofectamine 2000 according to the protocol of the manufacturer. The medium was then renewed and treatments were started. After treatment, the cell lysates were subjected to the dual luciferase reporter assay (Promega) according to the recommendations of the manufacturer and luciferase activities were measured with the GloMax-20/20 luminometer (Promega). The results were expressed as relative luciferase activity, which is the ratio of firefly luciferase activity to renilla luciferase activity.

### MMP2 mRNA stability assay

Cells were pretreated with PP2A inhibitors for 24 hours and transcription was then blocked with 5.0 μg/ml actinomycin D (ActD) for 3 hours in the presence of PP2A inhibitors. mRNA was then harvested at different time intervals (0, 6, 12, or 24 hours) to test MMP2 mRNA expression by using real-time PCR.

### Microarray assay

Sample preparation and processing procedure were performed as described in detail in the Agilent GeneChip Expression Analysis Manual (Santa Clara, CA). Differentially expressed genes were screened using Agilent 44K human whole-genome oligonucleotide microarrays. The selection criterion was defined as a more than 1.5-fold difference in the level of expression (difference in upregulated expression more than 1.5-fold, and difference in downregulated expression less than 0.67-fold). Hierarchical clustering of samples was done by average linkage algorithm using TIGR MultiExperiment Viewer (The Institute for Genomic Research, Rockville, MD, USA). Kyoto Encyclopedia of Genes and Genomes (KEGG) pathway database (http://www.genome.jp/kegg/pathway.html) was used for pathway analysis and mapping^9^.

### Statistical analysis

Each experiment was performed at least in triplicate. Results were expressed as the mean value ± standard deviation (SD). Statistical analysis was performed using an unpaired Student’s t-test. A *P* value of less than 0.05 was considered significant.

## Results

### Cantharidin repressed the invasive ability and MMP2 expression in pancreatic cancer cells

**Figure 1:**
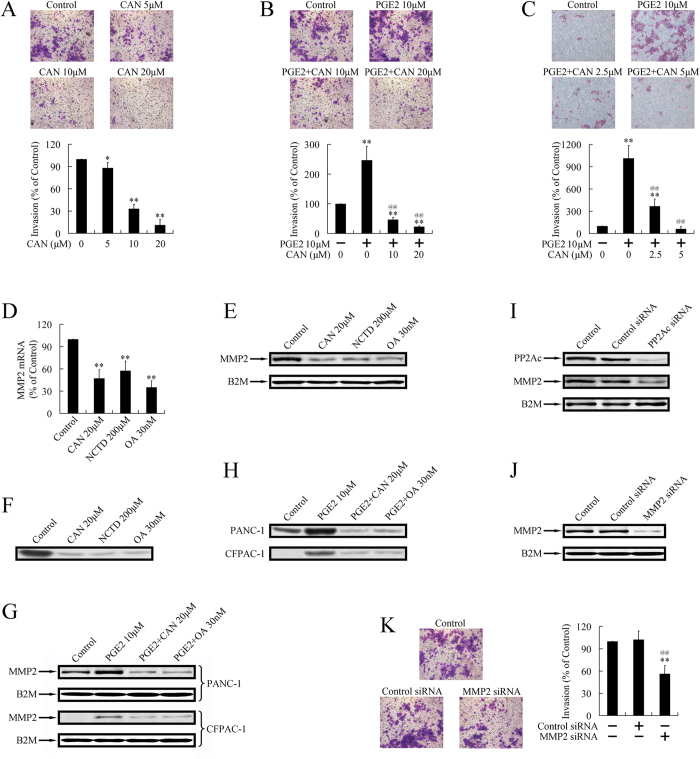
PP2A inhibitors repressed invasion and MMP2 expression in pancreatic cancer cells. (**A**) Cantharidin (CAN) dose-dependently repressed the invasive ability of PANC-1 cells. *P < 0.05 and **P < 0.01 indicates significant differences from control group. PGE2 promoted invasion of PANC-1 cells (**B**) and CFPAC-1 cells (**C**). Cantharidin dose-dependently repressed the PGE2-driven invasion in PANC-1 cells (**B**) and CFPAC-1 cells (**C**). **P < 0.01 indicates significant differences from control group. ^@@^*P *< 0.01 indicates significant differences from PGE2 group. (**D**). Treatment with PP2A inhibitors, cantharidin, norcantharidin, and Okadaic acid (OA), for 24  repressed the expression of MMP2 at the mRNA level. **P < 0.01 indicates significant differences from control group. (**E**). Treatment with PP2A inhibitors for 24 h repressed the expression of MMP2 at the protein level. The gels have been run under the same experimental conditions. (**F**). Zymography verified that treatment with PP2A inhibitors for 24 h repressed activity of MMP2. (**G**). Treatment with PP2A inhibitors, cantharidin, and OA for 24 h repressed PGE2-stimulated expression of MMP2. (**H**). Zymography verified that treatment with PP2A inhibitors, cantharidin, and OA for 24 h repressed PGE2-stimulated activation of MMP2. (**I**). PP2Ac targeted siRNA repressed expression of PP2Ac and MMP2. (**J**). MMP2 targeted siRNA repressed expression of MMP2. (**K**). MMP2-siRNA inhibited invasion of pancreatic cancer cells. **P < 0.01 indicates significant differences from control group. ^@@^*P *< 0.01 indicates significant differences from control-siRNA group.

Effects of cantharidin on the invasion of pancreatic cancer cells were evaluated by transwell assay. As shown in Fig. 1A, treatment with cantharidin dose-dependently repressed the invasive ability of PANC-1 cells. Inflammatory factor PGE2 promoted invasion of pancreatic cancer cells, which could also be repressed by cantharidin (Fig. 1B,C).

Down-regulated MMP2 mRNA level upon cantharidin treatment was shown by real-time PCR (Fig. 1D). Norcantharidin, a derivative of cantharidin, and OA, a classical PP2A inhibitor, could also repress the expression of MMP2 (Fig. 1D). The downregulation of MMP2 at the protein level was further confirmed by western blot (Fig. 1E). The downregulated MMP2 activity upon treatment with PP2A inhibitors was also observed by zymography (Fig. 1F). Besides the repression on basal expression and activity of MMP2, PP2A inhibitors could also decrease the PGE2-stimulated MMP2 expression and activation (Fig. 1F,G).

**Figure 2:**
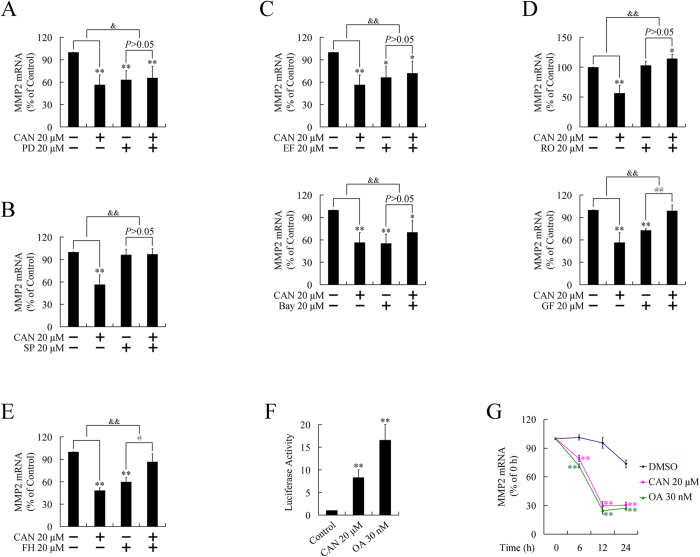
Transcriptional regulation of MMP2 by PP2A inhibitors. (**A**)–(**E**). Participation of ERK, JNK, PKC, NF-κB, and β-catenin pathways in the repression of MMP2 by cantharidin (CAN). Down-regulation of MMP2 could be attenuated or reversed by pretreatment with ERK pathway inhibitor PD98059 (PD) (**A**), JNK inhibitor SP600125 (SP) (**B**), PKC inhibitors GF109203X (GF) or RO31-8220 (RO), (**C**), NF-κB pathway inhibitors EF-24 (EF) or Bay11-7082 (Bay), (**D**), and β-catenin pathway inhibitor FH535 (FH) (E). *P < 0.05, **P < 0.01 indicates significant differences from respective control groups; ^@^*P* < 0.05, ^@@^*P* < 0.01 indicates significant differences from cell signaling pathway inhibitor groups^; &^*P* < 0.05, ^&&^*P* < 0.01 indicates significant differences between folds induction. (**F**). Transcriptional activity of MMP2 promoter after treatment with PP2A inhibitors, cantharidin, or OA for 24 h. **P < 0.01 indicates significant differences from control group. (**G**). Degradation of MMP2 mRNA after treatment with PP2A inhibitors, cantharidin, or OA. **P < 0.01 indicates significant differences from control group.

Previous studies proved that cantharidin, norcantharidin, and OA presented to be selective, but not specific inhibitors of PP2A^10^. Therefore, we used siRNA targeting PP2Acα (α isoform of PP2Ac), to create a specific inhibition of PP2A. Knockdown of PP2Ac, as well as downregulation of MMP2, was confirmed by western blot (Fig. 2F), suggesting the repression of MMP2 by cantharidin, norcantharidin, and OA could be executed through inhibition of PP2A.

To verify whether downregulation of MMP2 could further result in repressed invasion, we used siRNAs targeting MMP2, and tested cell invasive ability using transwell assay. As shown in Fig. 1J,K, knockdown of MMP2 led to repressed invasion, suggesting the repression on cell invasion by PP2A inhibitors could be due to repression of MMP2.

### Cantharidin repressed MMP2 expression through multiple pathways involving RNA degradation

In our previous studies^3^,^4^,^5^,^6^, we found that ERK, JNK, NF-κB, and PKC pathways^3^,^4^,^5^ were activated, while the WNT/β-catenin pathway was repressed following treatment with PP2A inhibitors^6^. Therefore, we investigated whether these pathways were involved in the downregulation of MMP2.

Pretreatment with ERK pathway inhibitor (PD98059), JNK inhibitor (SP600125), PKC inhibitors (GF109203X or RO31-8220), NF-κB pathway inhibitors (EF-24, the inhibitor of IKK, or Bay 11-7082, the inhibitor of IκB), or β-catenin pathway inhibitor (FH535) attenuated the downregulation of MMP2 (Fig. 2A–E), suggesting all these pathways participated in the repression on MMP2 by cantharidin.

To investigate whether the downregulation of MMP2 occurred at the transcription level, we performed luciferase report gene assays. Interestingly, transcriptional activity of MMP2 was significantly increased, but not repressed, after treatment with PP2A inhibitors (Fig. 2F), suggesting a post-transcriptional control mechanism might be involved. This hypothesis was further confirmed by using a RNA stability assay. Treatment with PP2A inhibitors resulted in a significantly accelerated degradation of MMP2 mRNA (Fig. 2G), indicating that the downregulation of MMP2 was executed post-transcriptionally.

### PP2A inhibitors upregulated multiple genes involved in RNA degradation

**Figure 3:**
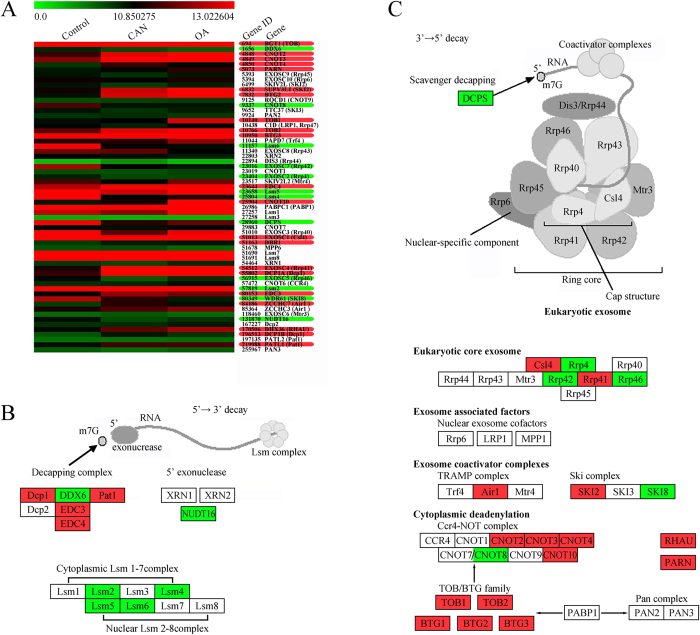
Identification of genes involved in RNA degradation after treatment with 20 μM cantharidin (CAN) or 30 nM OA for 24 h. (**A**). Illustration of the microarray results. (**B**). Classification analysis of genes involved in 5′→3′ decay pathway. (**C**). Classification analysis of genes involved in 3′→5′ decay pathway. Up-regulated genes are highlighted in red, and downregulated genes are highlighted in green. Pathway analysis and mapping was performed by using Kyoto Encyclopedia of Genes and Genomes (KEGG) pathway database (http://www.genome.jp/kegg/pathway.html).

To investigate the detailed mechanisms involved in MMP2 degradation, we performed microarray analyses to determine the mRNA expression changes of genes involved in RNA degradation regulation. Genes that significantly changed expression levels (1.5-fold) in both cantharidin and OA treated groups were chosen for further analysis. Of the 61 genes analyzed, 33 fulfilled this criterion (Fig. 3 CA). Although some genes participating in the 5′→3′ decay pathway were downregulated (Fig. 3B), a number of genes involved in the 3′→5′ decay pathway were upregulated (Fig. 3C), including 2 components of eukaryotic core exosome (Csl4 and Rrp41), 2 genes belonging to exosome coactivator complexes (Air1 and SKI2), and 11 genes participating in cytoplasmic deadenylation (*PARN*, *RHAU*, *CNOT2*, *CNOT3*, *CNOT4*, *CNOT10*, *TOB1*, *TOB2*, *BTG1*, *BTG2* and *BTG3*). Among the cytoplasmic deadenylation-related genes were *CNOT2, CNOT3, CNOT4, CNOT10* as well as other components from the Ccr4-NOT complex. TOB1, TOB2, BTG1, BTG1, BTG2 and BTG3 belong to TOB/BTG family, which enhance the deadenylase activity of the Ccr4-NOT complex. All Ccr4-NOT complexes containing either CNOT7 or CNOT8, which serve as a bridge to link the TOB/BTG family and the Ccr4-NOT complex^11^,^12^. Additionally, two other important deadenylation-related genes, Poly(A)-specific RNase (*PARN*) and RNA helicase associated with AU-rich element (*RHAU*)^13^,^14^, were found to be upregulated.

### PP2A inhibitors promoted MMP2 mRNA degradation through the cytoplasmic deadenylation pathway

**Figure 4:**
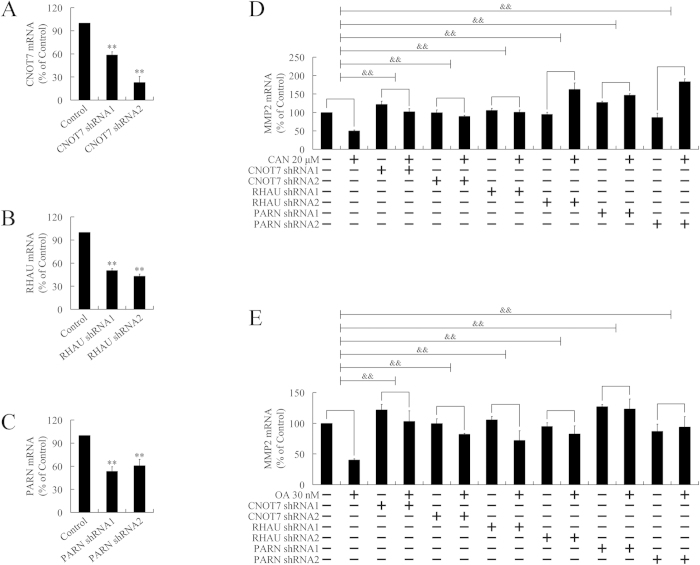
Participation of cytoplasmic deadenylation in MMP2 mRNA degradation. (**A**)–(**C**). Confirmation of knockdown of CNOT7 (A), RHAU (**B**), and PARN (**C**) by lentivirus-mediated shRNAs by using real-time PCR. **P < 0.01 indicates significant differences from respective control groups. (**D**) and (**E**). Participation of cytoplasmic deadenylation in MMP2 mRNA degradation. CNOT7, RHAU, and PARN were interfered, respectively, in PANC-1 cells by lentivirus-mediated shRNAs, followed by treatment with 20 μM cantharidin (CAN) (**D**) or 30 nM OA (**E**) for 24 h. ^&&^*P* < 0.01 indicates significant differences between folds induction.

To verify whether a cytoplasmic deadenylation mechanism was involved in the accelerated degradation of MMP2, CONT7, PARN, and RHAU were knockdown by respective lentivirus-mediated shRNA. Among these three targets, PARN and RHAU were upregulated by PP2A inhibitors. Although expression of CONT7 was unchanged by PP2A inhibitors, CONT7 is critical for the assembling of the Ccr4-NOT complex and the recruitment of TOB/BTG family members, especially when CNOT8 was found to be downregulated in the present microarray assay. Knockdown of CNOT7, RHAU, or PARN was confirmed by real-time PCR (Fig. 4A–C). Cells were treated with PP2A inhibitors and expression of MMP2 was evaluated. As shown in Fig. 4D,E, silencing of CNOT7, RHAU, and PARN attenuated or reversed the downregulation of MMP2 by PP2A inhibitors, suggesting cantharidin and OA might accelerate degradation of MMP2 mRNA through a cytoplasmic deadenylation-dependent manner.

### Cantharidin upregulated deadenylation-related genes through multiple pathways

**Figure 5:**
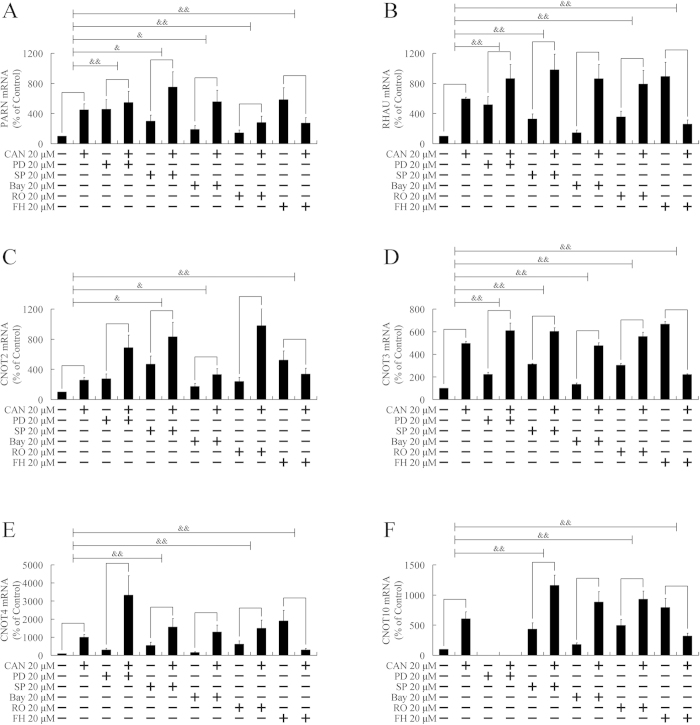
Participation of ERK, JNK, PKC, NF-κB, and β-catenin pathways in upregulation of PARN, RHAU, CNOT2, CNOT3, CNOT4, and CNOT10. PANC-1 cells were pretreated with ERK pathway inhibitor (PD98059, PD), JNK inhibitor (SP600125, SP), NF-κB pathway inhibitor (Bay11-7082, Bay), PKC inhibitors (RO31-8220, RO), or β-catenin pathway inhibitor (FH535, FH) for 3 h, followed by treatment with 20 μM cantharidin (CAN) for 24 h. Expressions of PARN (**A**), RHAU (**B**), CNOT2 (C), CNOT3 (**D**), CNOT4 (**E**), and CNOT10 (**F**) were evaluated by real-time PCR. ^&^*P* < 0.05, ^&&^*P* < 0.01 indicates significant differences between fold induction.

**Figure 6:**
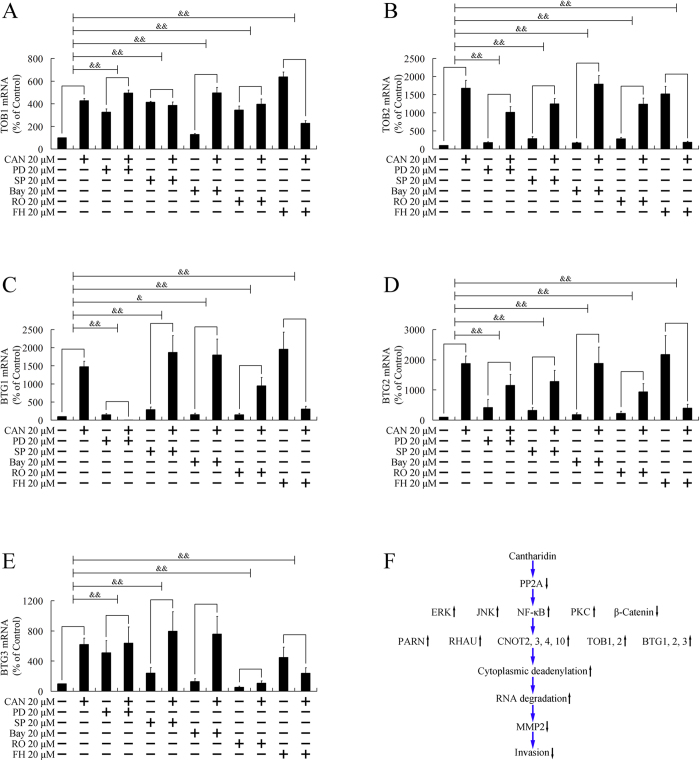
Participation of ERK, JNK, PKC, NF-κB, and β-catenin pathways in upregulation of TOB1, TOB2, BTG1, BTG2, and BTG3. PANC-1 cells were pretreated with ERK pathway inhibitor (PD98059, PD), JNK inhibitor (SP600125, SP), NF-κB pathway inhibitor (Bay11-7082, Bay), PKC inhibitors (RO31-8220, RO), or β-catenin pathway inhibitor (FH535, FH) for 3 h, followed by treatment with 20 μM cantharidin (CAN) for 24 h. Expressions of TOB1 (**A**), TOB2 (B), BTG1 (**C**), BTG2 (**D**), and BTG3 (**E**) were evaluated by real-time PCR. ^&^*P* < 0.05, ^&&^*P* < 0.01 indicates significant differences between fold induction. (**F**). Cell signaling transduction mechanism involved in the present study.

The above paragraphs demonstrated that multiple cell signaling pathways are involved in the degradation of MMP2, including ERK, JNK, PKC, NF-κB, and β-catenin pathways. Next, we investigated whether these pathways were involved in the upregulation of PARN, RHAU, CNOT2, CNOT3, CNOT4, CNOT10, TOB1, TOB2, BTG1, BTG2, and BTG3. As shown in Figs 5,6, pretreatment with PD98059, an ERK pathway inhibitor, attenuated the upregulation of PARN, RHAU, CNOT3, TOB1, TOB2, BTG1, BTG2, and BTG3. SP600125, a JNK inhibitor, and FH535, a β-catenin pathway inhibitor, repressed the elevation of all 11 genes. Bay11-7082, the inhibitor of the NF-κB pathway, impaired the increase of PARN, CNOT2, CNOT3, TOB1, TOB2, BTG1, and BTG2. RO31-8220, a PKC inhibitor, attenuated the upregulation of PARN, RHAU, CNOT3, CNOT4, CNOT10, TOB1, TOB2, BTG1, BTG2, and BTG3. As multiple pathways were involved in the upregulation of deadenylation-related genes, therefore, multiple pathways were involved in the downregulation of MMP2.

## Discussion

Pancreatic cancer is characterized by local invasion, early metastasis, and a strong desmoplastic reaction. *In vitro* studies have demonstrated that proteolytic degradation of extracellular matrix (ECM) components is a major step in pancreatic cancer invasion^15^,^16^. MMPs are a family of ECM modifying enzymes associated with tumour progression and metastasis by degradation of all components of the ECM. Since the 72 kDa type IV collagenase (MMP2) degrades collagen IV, one of the major components of the basement membrane, it is thought to be of special significance during tumour invasion. Indeed, previous studies have supported the role of MMP2 in basement membrane degradation during the invasion of pancreatic cancer cells^15^,^16^.

A previous study found that MMP2 was downregulated by treatment with cantharidin in bladder carcinoma cells^7^. In this previous study, phosphorylation levels of p38 and JNK, two pathways upstream of MMP2, were repressed by cantharidin^7^. In addition, the inhibition of these two pathways was thought to be responsible for the downregulation of MMP2^7^. However, in our previous study, phosphorylation levels of p38 and JNK were elevated by cantharidin in pancreatic cancer cells^3^, indicating cantharidin might downregulate MMP2 in pancreatic cancer cells through a manner different from that in bladder cancer cells.

Mechanistically, cantharidin is an inhibitor of PP2A, which is the main negative regulator of multiple kinases, including JNK, ERK, PKC, and IKK. Therefore, treatment with cantharidin should result in phosphorylation of these kinases. In our previous studies, we did find that treatment with cantharidin led to phosphorylation and activation of these kinases in pancreatic cancer cells. In fact, in the present study, inhibitors of these kinases attenuated the downregulation of MMP2 by cantharidin, suggesting activation of all these kinase pathways were involved in the repression of MMP2.

Pancreatic cancer is an inflammation-driven neoplasm. Epidemiological and experimental data have demonstrated close relationships between chronic pancreatitis and pancreatic cancer^17^,^18^. In recent years, many efforts have been given to identify the underlying mechanisms. Elevated interleukin-1 (IL-1) and transforming growth factor β (TGF-β) levels have been found in the serum of pancreatic cancer patients^19^,^20^. IL-1α has been shown to promote proliferation, adhesion, and migration of pancreatic cancer cell lines, and is associated with the activation of Ras and the downstream ERK signaling pathway^21^. However, IL-1α activated NF-κB pathway and upregulated several anti-apoptotic genes, such as BcL-XL and BfL-1^22^. TGF-β upregulates MMP2 and the uPA system and, thus, strengthens invasive ability^23^. IL-8, as well as IL-6, can activate the Mitogen-activated protein kinase (MAPK) pathway, leading to production of vascular endothelial growth factor (VEGF) and neuropilin-2^24^,^25^, both of which have been correlated with angiogenesis and metastatic behaviour of pancreatic cancer cells^24^,^25^,^26^. Many cancers that overexpress cyclooxygenase-2 (COX-2) have been shown to have high intratumoral levels of PGE2, which has been shown to upregulate the invasive potential of pancreatic cancer cells through an ERK/Ets-1-dependent induction of MMP-2 expression and activity^15^.

Consistent with activated ERK pathway after cantharidin treatment, transcriptional activation of MMP2 was observed upon treatment with PP2A inhibitors. This increased transcription and decreased expression highly suggests the involvement of a post-transcriptional regulation mechanism. Not surprisingly, by using a RNA stability assay, we showed accelerated MMP2 mRNA degradation. Thus, in the current study, we found that PP2A inhibitors repressed expression of MMP2 post-transcriptionally but not at the transcription level. As inflammation promotes MMP2 expression through transcriptional activation^15^, the present study may explain why inflammation-stimulated pancreatic cancer invasion could be repressed by PP2A inhibitors.

Post-transcriptional regulation is increasingly recognized as a critical control point in gene expression. The mRNAs in a eukaryotic cell have a wide range of half-lives. Modulation of mRNA stability can affect the steady-state levels of transcripts without changes in transcription rate, permitting rapid responses to metabolic or cell cycle events^27^. Almost all eukaryotic mRNAs contain a typical boundary cap structure at the 5′-end and a poly (A) tail at the 3′ end, and these modifications are crucial for mRNA processing, transportation, efficient translation, and stability^28^. The removal of the poly (A) tail, a process termed as deadenylation, renders transcripts translationally silent and is the first step in the decay of the majority of transcripts in eukaryotic cells^29^. Thereafter, the 5′-cap structure is removed and the remaining portion of the mRNA is rapidly degraded^11^. Thus, deadenylation is often the first and rate-limiting step for mRNA decay and translational silencing, and has the ability to profoundly influence cellular gene expression on multiple levels^29^,^30^. By using microarray analyses, we evaluated the expression of several genes involved in RNA degradation. Among the investigated 61 genes, 12 genes were downregulated and 20 genes were upregulated. Further classification analysis explored that 11 of these upregulated genes participated in cytoplasmic deadenylation, the initiative step of mRNA decay, suggesting that elevated deadenylation might be the critical mechanism involved in accelerated MMP2 degradation by PP2A inhibitors.

Deadenylation is catalysed by a specific family of exoribonucleases, known as deadenylases^29^,^30^, which are organized in complexes, such as the carbon catabolite repressor 4-negative on TATA or CNOT (Ccr4-NOT) and the poly (A)-nuclease (PAN2/PAN3) complex, or may form homodimers, such as Poly (A)-specific RNase (PARN)^30^. The Ccr4-NOT complex is highly conserved, multifunctional machinery that controls mRNA metabolism. Its components have been implicated in several aspects of mRNA and protein expression, including transcription initiation, elongation, mRNA degradation, ubiquitination, and protein modification^12^,^29^. Distinct Ccr4-NOT complexes contain either CNOT7 or CNOT8. CNOT7 and CNOT8 may compete for the same binding site on the scaffold protein CNOT1 and can compensate for each other’s function, which is to be expected given their high amino acid sequence similarity^12^. In the present study, although the expression of CNOT8 was downregulated, thepresence of CNOT7 might be a supplement, and promoted MMP2 mRNA degradation in company with elevated CNOT2, CNOT3, CNOT4, and CNOT10.

The TOB/BTG family consists of TOB1, TOB2, BTG1, BTG2/Tis21/PC3, PC3B, and BTG3/ANA in mammalian cells^11^,^12^. Recent reports show that TOB/BTG family members enhanced the deadenylase activity of the Ccr4-NOT complex through binding to CNOT7. Therefore, CNOT7 serves as a bridge to link the TOB/BTG family and the Ccr4-NOT complex rather than as a deadenylase^11^,^12^. Considering the central role that CNOT7 possesses, we used shRNA to target CNOT7 to block the assembling of the Ccr4-NOT complex and to break the bridge linking the TOB/BTG family to the Ccr4-NOT complex.

PARN is a key deadenylase involved in regulating gene expression in mammals and is an enzyme with homology to the RNase D family^13^,^27^. PARN initiates decay of mRNAs containing AU-rich elements (ARE) or nonsense codons. *In vivo* and *in vitro* studies have shown that RNA helicase associated with AU-rich element (RHAU) enhances poly (A) shortening and decay of mRNA in a manner dependent on ARE in the message and ATPase activity of RHAU. RHAU interacts with PARN and the exosome, indicating that mRNA decay is triggered by RHAU recruitment of the RNA-degrading machinery to the ARE in the mRNA^14^.

In the present study, knockdown of CNOT7, PARN, or RHAU by targeting shRNA attenuated the repression of MMP2 by cantharidin, confirming the participation of cytoplasmic deadenylation in elevated MMP2 degradation. In our previous studies, we found that treatment with cantharidin induced inhibition of PP2A, leading to phosphorylation of multiple substrates, including ERK, JNK, PKC, IKK, and β-catenin. By using real-time PCR, we presently found that JNK, ERK, PKC, NF-κB, and β-catenin pathways were involved in the upregulation of PARN, RHAU, CNOT2, CNOT3, CNOT4, CNOT10, TOB1, TOB2, BTG1, BTG2, and BTG3, consistent with the multiple pathway-involved MMP2 degradation.

Of note, members of the TOB/BTG family present with antiproliferative activity^12^ and PARN may play an important role in the degradation of cancer-related mRNAs, such as IL-8 and VEGF mRNAs, and potentially act as a tumour suppressor^30^. Elevation of these genes by cantharidin might not only affect the invasion, but also other cell-biological behaviours of pancreatic cancer cells. In our previous studies, we have proven the inhibition on proliferation and migration, as well as induction of apoptosis upon treatment of cantharidin in pancreatic cancer cells. Whether or not the mRNA degradation mechanism is involved in these anticancer effects still needs further investigation.

Taken together, our recent and present investigations have explored the mechanisms involved in the anti-cancer effect of cantharidin, including NF-κB pathway mediated apoptosis induction^4^, JNK pathway-dependent growth inhibition^3^,^5^, Wnt/β-catenin pathway mediated inhibition on migration^6^, and multiple pathway-involved degradation of MMP2, leading to repressed invasive behaviour. Our findings provide convincing evidence for how cantharidin, as well as other PP2A inhibitors, carry out anti-tumour effects and reveal the possibility of using PP2A as a therapeutic target for pancreatic cancer treatment.

## Additional Information

**How to cite this article**: Shen, M. *et al*. Cantharidin represses invasion of pancreatic cancer cells through accelerated degradation of MMP2 mRNA. *Sci. Rep*. **5**, 11836; doi: 10.1038/srep11836 (2015).

## Figures and Tables

**Table 1:** Primers.

Genes	Sense(5′−3′)	Antisense(3′−5′)	Pruduct size (bp)
MMP2	TTCCGCTTCCAGGGCACA	CACCTTCTGAGTTCCCACCAA	157
CNOT2	GCCATCCACCTTCAACTACAACC	AAATTCAGACCCTGCTCAAACTAG	281
CNOT3	AGAAGAAGGGCGACAAGGATAAG	ATGGCGTCAACGAGGATGG	136
CNOT4	TCGGTGGTTTCTTGTGAGGAC	CATCTATCTCCAAGGGCTCCAT	235
CNOT7	GTTGCTATGGACACCGAGTTTC	CAAGTTGAAGTTCCTGGAGGG	176
CNOT10	TGCCATGAGCAAGCACAATT	GTAGCCAGAGGCGAGGATTT	269
TOB1	ACTTCTCCCATCAACCTCGG	CCAAGCCAAGCCCATACAG	94
TOB2	CCCGCCTGATGTATTGGAT	GCTTTGGTGCTTTCTGGTGA	189
BTG1	CGGGTTACCGTTGTATTCGC	ATTCGGCTGTCTACCATTTGC	247
BTG2	TGTGAGCCAGTGTCTGCCTAT	CTAACCTTGCTTGCTCCCTCT	119
BTG3	GAAATTGCTGCCGTTGTCTT	TTTTCACAGGCTTTCAGGACAT	224
PARN	AATCGGTTGTGCCTCCCTG	CGCCACGCATAAGAACAGAAGT	224
RHAU	AACTTTCCGCCACGATTCC	TTGTTCATGTCCCAGGGTTTG	134
B2M	TCAAGAAGGTGGTGAAGCAG	AAGGTGGAGGAGTGGGTGTC	112

## References

[b1] Wolfgang C. L. . Recent progress in pancreatic cancer. CA Cancer J Clin 63, 318–48 (2013). 23856911 10.3322/caac.21190 PMC3769458

[b2] Paulson A. S., Tran Cao H. S., Tempero M. A. & Lowy A. M. Therapeutic advances in pancreatic cancer. Gastroenterology 144, 1316–26 (2013). 23622141 10.1053/j.gastro.2013.01.078

[b3] Li W. . Cantharidin, a potent and selective PP2A inhibitor, induces an oxidative stress-independent growth inhibition of pancreatic cancer cells through G2/M cell-cycle arrest and apoptosis. Cancer Sci 101, 1226–33 (2010). 20331621 10.1111/j.1349-7006.2010.01523.x PMC11158714

[b4] Li W. . PP2A inhibitors induce apoptosis in pancreatic cancer cell line PANC-1 through persistent phosphorylation of IKKalpha and sustained activation of the NF-kappaB pathway. Cancer Lett 304, 117–27 (2011). 21376459 10.1016/j.canlet.2011.02.009

[b5] Li W. . Growth of the pancreatic cancer cell line PANC-1 is inhibited by protein phosphatase 2A inhibitors through overactivation of the c-Jun N-terminal kinase pathway. Eur J Cancer 47, 2654–64 (2011). 21958460 10.1016/j.ejca.2011.08.014

[b6] Wu M. Y. . PP2A inhibitors suppress migration and growth of PANC-1 pancreatic cancer cells through inhibition on the Wnt/beta-catenin pathway by phosphorylation and degradation of beta-catenin. Oncol Rep 32, 513–22 (2014). 24926961 10.3892/or.2014.3266 PMC4091883

[b7] Huang Y. P. . Suppressions of Migration and Invasion by Cantharidin in TSGH-8301 Human Bladder Carcinoma Cells through the Inhibitions of Matrix Metalloproteinase-2/-9 Signaling. Evid Based Complement Alternat Med 2013, 190281 (2013). 23431332 10.1155/2013/190281 PMC3568914

[b8] Bian J. & Sun Y. Transcriptional activation by p53 of the human type IV collagenase (gelatinase A or matrix metalloproteinase 2) promoter. Mol Cell Biol 17, 6330–8 (1997). 9343394 10.1128/mcb.17.11.6330 PMC232484

[b9] Du J. . KEGG-PATH: Kyoto encyclopedia of genes and genomes-based pathway analysis using a path analysis model. Mol Biosyst 10, 2441–7 (2014). 24994036 10.1039/c4mb00287c

[b10] Li Y. M. & Casida J. E. Cantharidin-binding protein: identification as protein phosphatase 2A. Proc Natl Acad Sci U S A 89, 11867–70 (1992). 1334551 10.1073/pnas.89.24.11867 PMC50658

[b11] Horiuchi M. . Structural basis for the antiproliferative activity of the Tob-hCaf1 complex. J Biol Chem 284, 13244–55 (2009). 19276069 10.1074/jbc.M809250200 PMC2676056

[b12] Bartlam M. & Yamamoto T. The structural basis for deadenylation by the CCR4-NOT complex. Protein Cell 1, 443–52 (2010). 21203959 10.1007/s13238-010-0060-8 PMC4875137

[b13] Utter C. J., Garcia S. A., Milone J. & Bellofatto V. PolyA-specific ribonuclease (PARN-1) function in stage-specific mRNA turnover in Trypanosoma brucei. Eukaryot Cell 10, 1230–40 (2011). 21743004 10.1128/EC.05097-11 PMC3187051

[b14] Iwamoto F., Stadler M., Chalupnikova K., Oakeley E. & Nagamine Y. Transcription-dependent nucleolar cap localization and possible nuclear function of DExH RNA helicase RHAU. Exp Cell Res 314, 1378–91 (2008). 18279852 10.1016/j.yexcr.2008.01.006

[b15] Ito H. . Prostaglandin E2 enhances pancreatic cancer invasiveness through an Ets-1-dependent induction of matrix metalloproteinase-2. Cancer Res 64, 7439–46 (2004). 15492268 10.1158/0008-5472.CAN-04-1177

[b16] Ellenrieder V. . Role of MT-MMPs and MMP-2 in pancreatic cancer progression. Int J Cancer 85, 14–20 (2000). 10585576 10.1002/(sici)1097-0215(20000101)85:1<14::aid-ijc3>3.0.co;2-o

[b17] Guerra C. . Chronic pancreatitis is essential for induction of pancreatic ductal adenocarcinoma by K-Ras oncogenes in adult mice. Cancer Cell 11, 291–302 (2007). 17349585 10.1016/j.ccr.2007.01.012

[b18] Lowenfels A. B. . Pancreatitis and the risk of pancreatic cancer. International Pancreatitis Study Group. N Engl J Med 328, 1433–7 (1993). 8479461 10.1056/NEJM199305203282001

[b19] Fukumura Y., Kumasaka T., Mitani K., Karita K. & Suda K. Expression of transforming growth factor beta1, beta2, and beta3 in chronic, cancer-associated, obstructive pancreatitis. Arch Pathol Lab Med 130, 356–61 (2006). 16519564 10.5858/2006-130-356-EOTGFA

[b20] Ren C., Chen Y., Han C., Fu D. & Chen H. Plasma interleukin-11 (IL-11) levels have diagnostic and prognostic roles in patients with pancreatic cancer. Tumour Biol (2014). 10.1007/s13277-014-2459-y 25123265

[b21] Mori A. . Up-regulation of Kruppel-like factor 5 in pancreatic cancer is promoted by interleukin-1beta signaling and hypoxia-inducible factor-1alpha. Mol Cancer Res 7, 1390–8 (2009). 19671674 10.1158/1541-7786.MCR-08-0525

[b22] Melisi D. . Secreted interleukin-1alpha induces a metastatic phenotype in pancreatic cancer by sustaining a constitutive activation of nuclear factor-kappaB. Mol Cancer Res 7, 624–33 (2009). 19435817 10.1158/1541-7786.MCR-08-0201 PMC2856954

[b23] Ellenrieder V. . TGF-beta-induced invasiveness of pancreatic cancer cells is mediated by matrix metalloproteinase-2 and the urokinase plasminogen activator system. Int J Cancer 93, 204–11 (2001). 11410867 10.1002/ijc.1330

[b24] Feurino L. W. . IL-6 stimulates Th2 type cytokine secretion and upregulates VEGF and NRP-1 expression in pancreatic cancer cells. Cancer Biol Ther 6, 1096–100 (2007). 17568185 10.4161/cbt.6.7.4328

[b25] Li M. . Interleukin-8 increases vascular endothelial growth factor and neuropilin expression and stimulates ERK activation in human pancreatic cancer. Cancer Sci 99, 733–7 (2008). 18307536 10.1111/j.1349-7006.2008.00740.x PMC2930017

[b26] Holmer R., Goumas F. A., Waetzig G. H., Rose-John S. & Kalthoff H. Interleukin-6: a villain in the drama of pancreatic cancer development and progression. Hepatobiliary Pancreat Dis Int 13, 371–80 (2014). 25100121 10.1016/s1499-3872(14)60259-9

[b27] Seal R., Temperley R., Wilusz J., Lightowlers R. N. & Chrzanowska-Lightowlers Z. M. Serum-deprivation stimulates cap-binding by PARN at the expense of eIF4E, consistent with the observed decrease in mRNA stability. Nucleic Acids Res 33, 376–87 (2005). 15653638 10.1093/nar/gki169 PMC546156

[b28] Zhang A., Liu W. F. & Yan Y. B. Role of the RRM domain in the activity, structure and stability of poly(A)-specific ribonuclease. Arch Biochem Biophys 461, 255–62 (2007). 17391638 10.1016/j.abb.2007.02.023

[b29] Lee J. E. . The PARN deadenylase targets a discrete set of mRNAs for decay and regulates cell motility in mouse myoblasts. PLoS Genet 8, e1002901 (2012). 22956911 10.1371/journal.pgen.1002901 PMC3431312

[b30] Maragozidis P. . Alterations of deadenylase expression in acute leukemias: evidence for poly(a)-specific ribonuclease as a potential biomarker. Acta Haematol 128, 39–46 (2012). 22614729 10.1159/000337418

