# Author Correction: Cantharidin represses invasion of pancreatic cancer cells through accelerated degradation of MMP2 mRNA

**DOI:** 10.1038/s41598-021-84394-4

**Published:** 2021-02-25

**Authors:** Meng Shen, Meng-Yao Wu, Long-Pei Chen, Qiaoming Zhi, Fei-Ran Gong, Kai Chen, Dao-Ming Li, Yadi Wu, Min Tao, Wei Li

**Affiliations:** 1 Department of Oncology, the First Affiliated Hospital of Soochow University, Suzhou, 215006 China; 2 Department of General Surgery, the First Affiliated Hospital of Soochow University, Suzhou, 215006 China; 3 Department of Hematology, the First Affiliated Hospital of Soochow University, Suzhou, 215006 China; 4 Jiangsu Institute of Clinical Immunology, Suzhou, 215006 China; 5 Institute of Medical Biotechnology, Soochow University, Suzhou, 215021 China; 6 PREMED Key Laboratory for Precision Medicine, Soochow University, Suzhou, 215021 China

Correction to: *Scientific Reports* https://doi.org/10.1038/srep11836, published online 02 July 2015

This Article contains errors in Figure 1. As a result of mistakes in figure assembly, the IHC images shown in the published Figure 1 are incorrect and do not correspond to the experiments described. The correct Figure 1 appears below as Figure 1.

**Figure 1:**
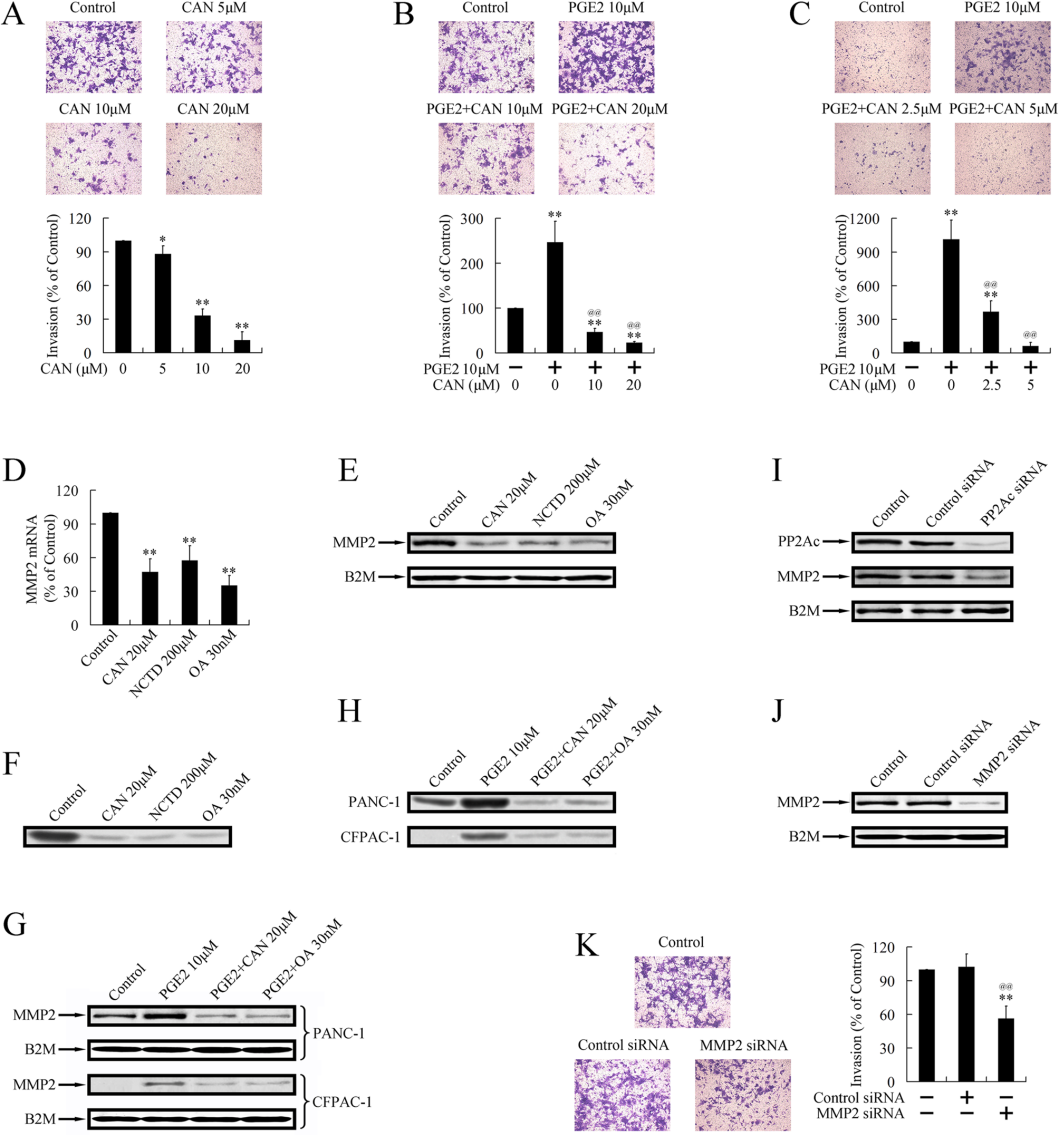
A correct version of the original Figure 1.

These changes do not affect the conclusions of the Article.

